# Supplementary material for: Humic Acids Affect the Detection of Metal Ions by Cyanobacteria Carbon Quantum Dots Differently
Source: Int J Environ Res Public Health. 2022 Aug 17;19(16):10225. doi: 10.3390/ijerph191610225 (PMC9408800; doi:10.3390/ijerph191610225)
Supplement: Supplementary file 1 [file ijerph-19-10225-s001.zip › ijerph-1865740-supplementary.pdf]

Supplementary Materials

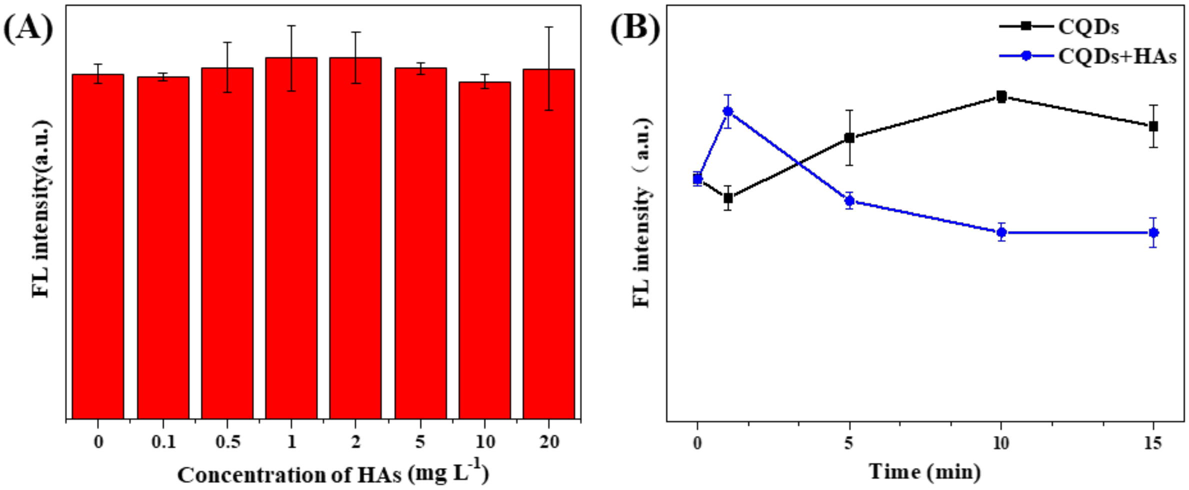

Figure S1. The fluorescence (FL) intensity of the cyanobacteria carbon quantum dots (CQDs) as affected by humic acids (HAs) depends on (**A**) the concentration of HAs and (**B**) the interaction time of CQDs with 10 mg L^{-1} of HAs.

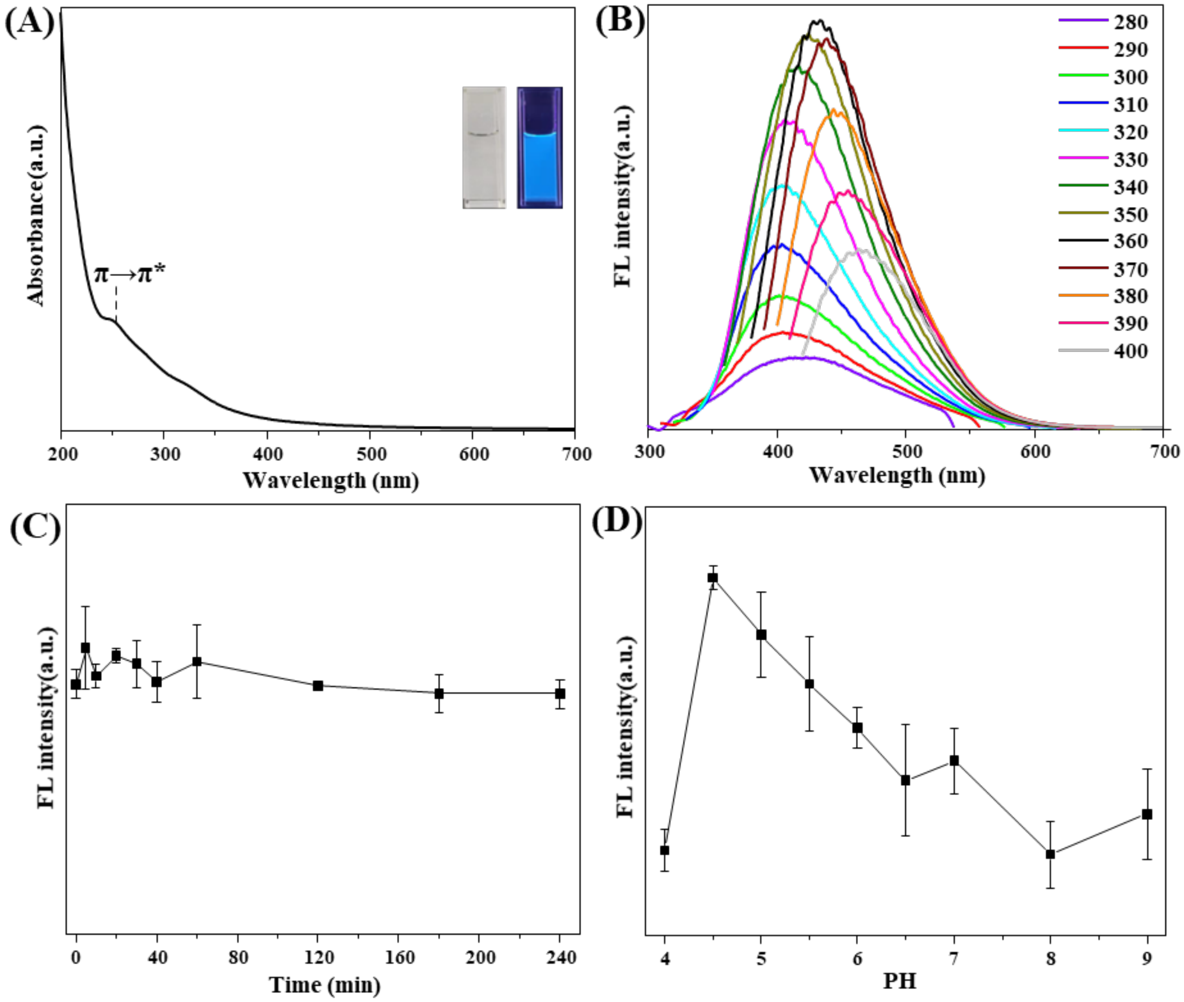

Figure S2. (**A**) Ultraviolet-visible (UV-vis) absorption spectra of the cyanobacteria carbon quantum dots (CQDs), (**B**) the fluorescence (FL) emission spectra of the CQDs with increasing excitation wavelengths from 280 to 400 nm in 10 nm increments, the stability of FL intensity in response to (**C**) time and (**D**) pH of the solution.

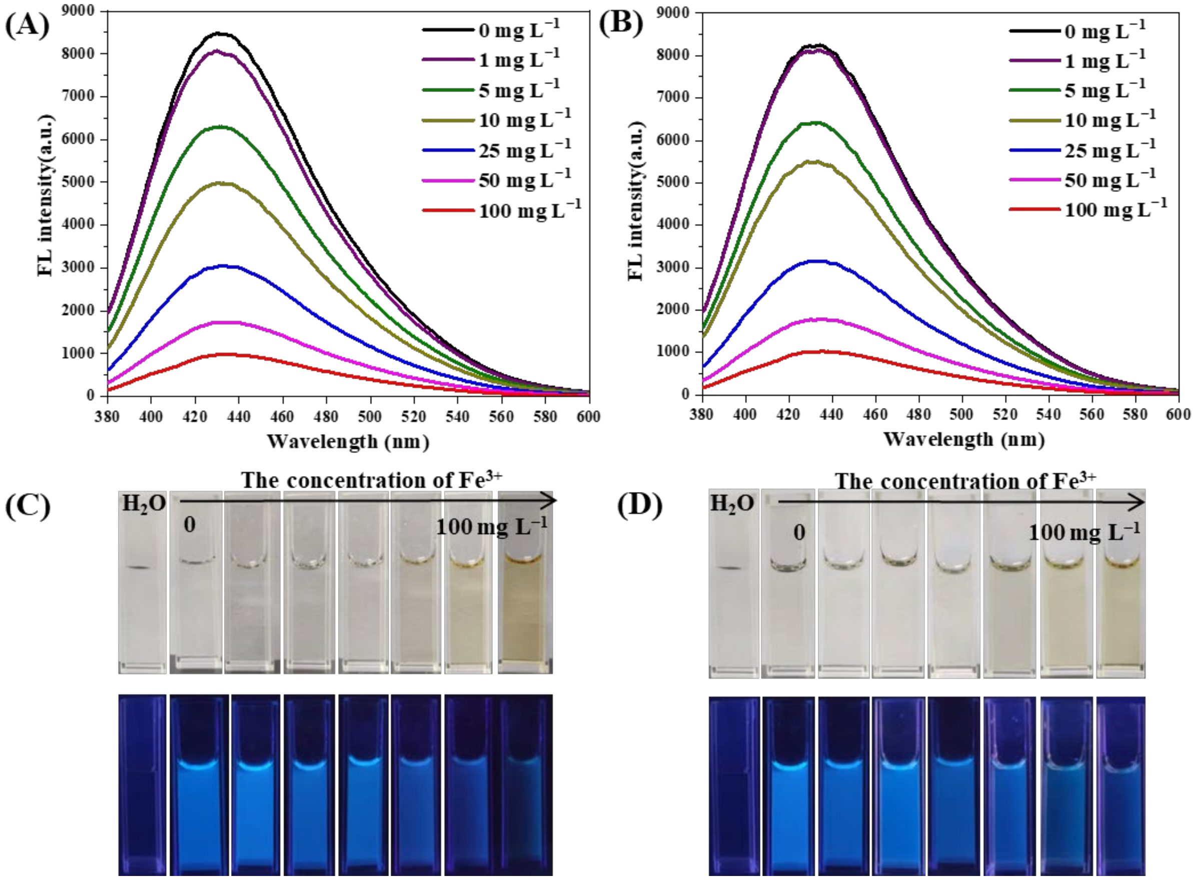

Figure S3. The effects of Fe^{3+} in the concentration in the range of 0 to 100 mg L^{-1} on the fluorescence (FL) intensity of (**A**) the cyanobacteria carbon quantum dots (CQDs) and (**B**) that with humic acids (HAs) coexisting; (**C**) the photos of the CQDs after interaction with different concentration of Fe^{3+} under sunlight (the first row) and ultraviolet light at 365 nm (the second row); (**D**) the photos of the CQDs with HAs coexisting after interaction with different concentration of Fe^{3+} under sunlight (the first row) and ultraviolet light at 365 nm (the second row).

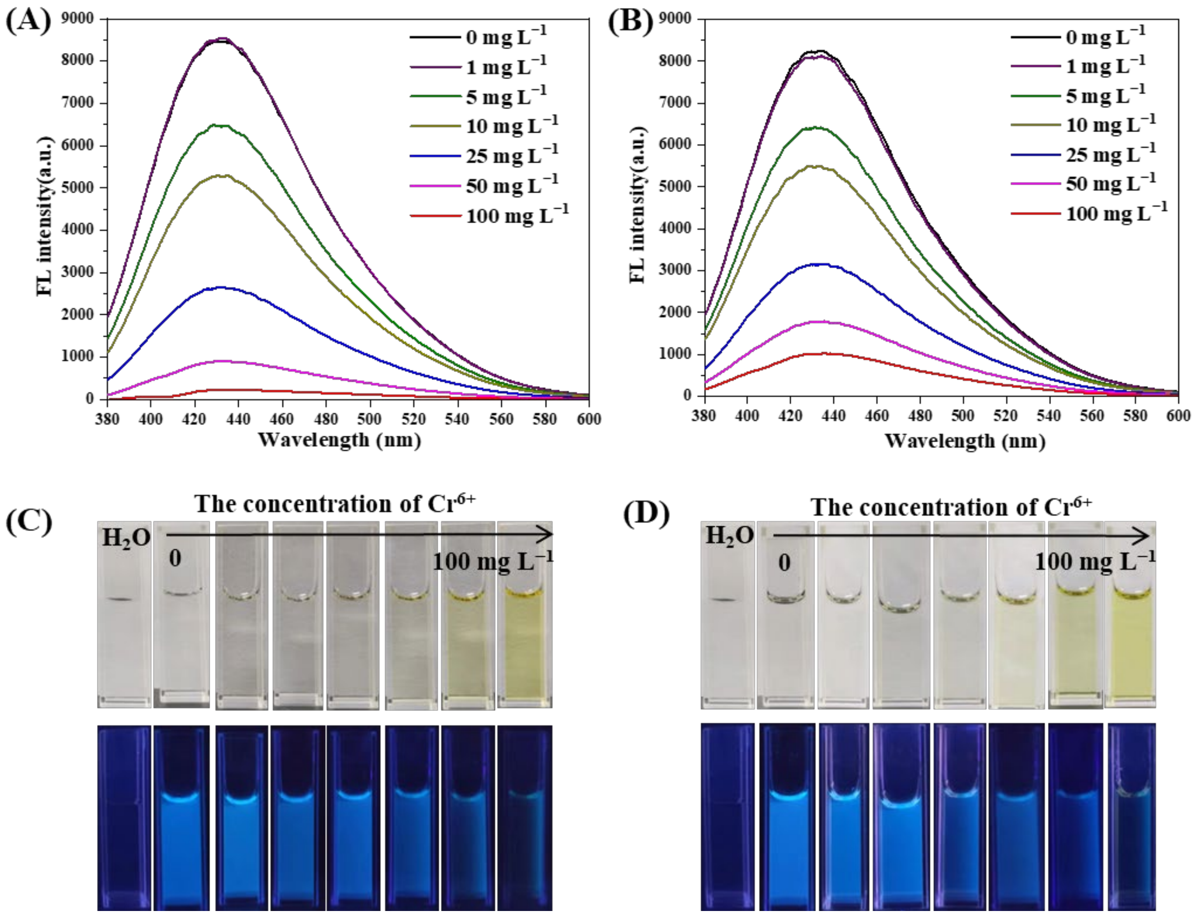

Figure S4. The effects of Cr^{6+} in the concentration in the range of 0 to 100 mg L^{-1} on the fluorescence (FL) intensity of (**A**) the cyanobacteria carbon quantum dots (CQDs) and (**B**) that with humic acids (HAs) coexisting; (**C**) the photos of the CQDs after interaction with different concentration of Cr^{6+} under sunlight (the first row) and ultraviolet light at 365 nm (the second row); (**D**) the photos of the CQDs with HAs coexisting after interaction with different concentration of Cr^{6+} under sunlight (the first row) and ultraviolet light at 365 nm (the second row).

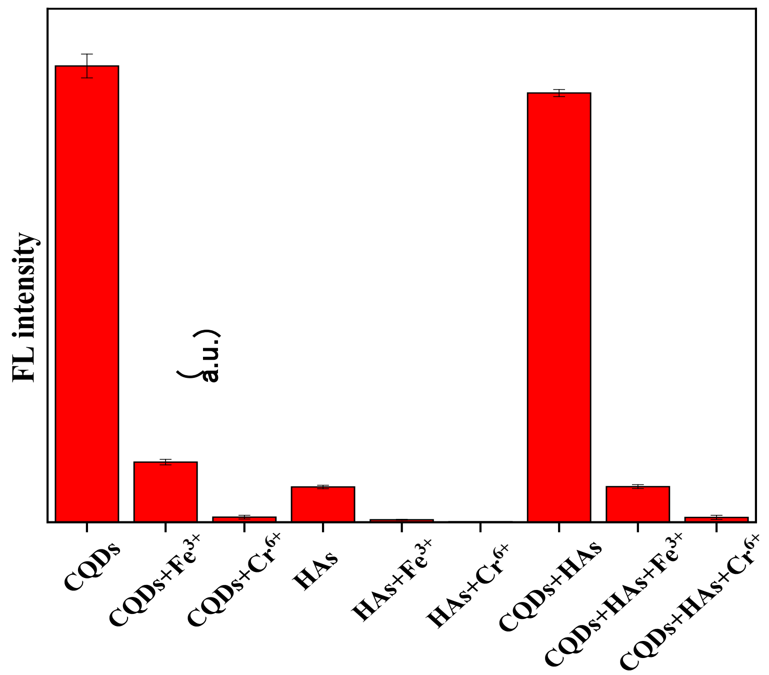

Figure S5 The fluorescence (FL) intensity of the cyanobacteria carbon quantum dots (CQDs) as affected by humic acids (HAs) or metal ions.

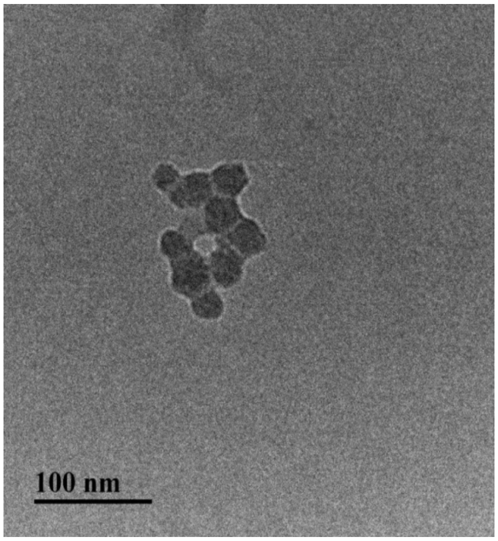

Figure S6 Transmission electron microscope image of the cyanobacteria carbon quantum dots as affected by humic acids .
